# Primary Progressive Aphasias: Diagnosis and Treatment

**DOI:** 10.3390/brainsci15030245

**Published:** 2025-02-25

**Authors:** Genaro Gabriel Ortiz, Héctor González-Usigli, Erick R. Nava-Escobar, Javier Ramírez-Jirano, Mario Alberto Mireles-Ramírez, Maribel Orozco-Barajas, Luis E. Becerra-Solano, Víctor J. Sánchez-González

**Affiliations:** 1 Department of Philosophical and Methodological Disciplines, and Molecular Biology Service in Medicine HC, University Health Sciences Center, University of Guadalajara, Guadalajara 44340, Jalisco, Mexico; genarogabriel@yahoo.com; 2 Department of Neurology, Sub-Specialty Medical Unit, Western National Medical Center, Mexican Institute of Social Security, Guadalajara 44329, Jalisco, Mexico; quincy1973@hotmail.com (H.G.-U.); erik_nava@outlook.com (E.R.N.-E.); dr_mireles@hotmail.com (M.A.M.-R.); 3 Neurosciences Division, Western Biomedical Research Center, Mexican Social Security Institute (IMSS), Guadalajara 44340, Jalisco, Mexico; ramirez_jirano@hotmail.com; 4 PhD Program on Biosciences, Health Department, Centro Universitario de Los Altos, University of Guadalajara, Tepatitlán de Morelos 47620, Jalisco, Mexico; maribel.orozco@academicos.udg.mx (M.O.-B.); luis.becerra@cualtos.udg.mx (L.E.B.-S.); 5 Clinical Department, Centro Universitario de Los Altos, University of Guadalajara, Tepatitlán de Morelos 47620, Jalisco, Mexico

**Keywords:** Primary Progressive Aphasia, Frontotemporal Lobar Degeneration, Alzheimer’s Disease, Neurodegeneration, Semantic Dementia

## Abstract

**Background and Objective:** Primary Progressive Aphasias (PPAs) are rare neurodegenerative disorders classified within frontotemporal lobar degeneration (FTLD) and typically manifest between 45 and 70 years of age. In Mexico—and many other countries—reliable epidemiological data are lacking; however, estimates suggest that PPA accounts for 0.5–2.5% of neurodegenerative disease cases in Memory Clinics, with an incidence of approximately 1 per 100,000 and an average survival of 8 years. This review aims to provide clinicians with an overview of PPA’s epidemiology, clinical features, and classification, thereby enhancing understanding of its subtypes and distinguishing characteristics from other aphasic conditions, such as vascular aphasia. **Methods:** This narrative review was conducted through a literature search using databases such as PubMed and Scopus. Relevant studies addressing the epidemiology, clinical presentation, and classification of PPA were identified, selected, and synthesized to offer a broad, clinically oriented overview of the condition. This approach was chosen to inform clinical practice and highlight the need for further targeted investigations, such as future systematic reviews focusing on specific aspects like therapeutic strategies. **Key Contents and Findings: (a) Epidemiology:** PPA is estimated to affect 0.5–2.5% of patients with neurodegenerative diseases in Memory Clinics, with an incidence of roughly 1 per 100,000. Average survival time is around 8 years (ranging from 3 to 17 years), with a generally balanced gender ratio, though some studies indicate a predominance of men. A positive family history is observed in 20–40% of cases, with about 10% following an autosomal dominant inheritance pattern. **(b) Clinical Characteristics and Classification:** PPA is marked by a gradual decline in language abilities, differentiating it from vascular aphasias. Subtypes include non-fluent forms (non-fluent progressive aphasia [nfPPA] and logopenic progressive aphasia [lPPA]), fluent forms (progressive fluent aphasia [PFA] and semantic dementia [SD]), and mixed forms (progressive mixed aphasia [PMA]). The neurodegenerative process in PPA extends beyond vascular boundaries, often resulting in presentations that deviate from classical Broca’s and Wernicke’s aphasias. Common symptoms include difficulties in word finding and naming, sometimes mistaken for memory loss, and, in the case of semantic dementia, personality changes that may go unnoticed by the patient. **Conclusions:** PPA is a heterogeneous and complex group of neurodegenerative disorders with significant clinical variability and a profound impact on patients and their families. While current epidemiological data are limited, this review emphasizes the need for further research to better delineate disease progression and refine diagnostic and therapeutic approaches. Future systematic reviews will be essential to address specific aspects of PPA, such as treatment strategies, to further improve patient care.

## 1. Introduction

Primary Progressive Aphasias (PPAs) are defined as language disorders characterized by an insidious onset and progressive evolution. The term “Primary Progressive Aphasia” applies only when these language impairments are the initial and predominant symptom, remaining isolated for an extended period. While other cognitive deficits may emerge as the disease advances, language difficulties remain the central and most significant feature throughout the progression [1].

In this narrative review, we conducted a comprehensive literature search to provide an overview of the current knowledge on Primary Progressive Aphasia (PPA) and its relationship with neurodegenerative diseases. Our approach involved the following steps:

1. Literature Search: We performed searches in major electronic databases such as PubMed and Scopus, using key terms related to PPA (e.g., “primary progressive aphasia”, “PPA”, “neurodegenerative diseases”) and specific subtopics of interest (e.g., “diagnosis”, “neuropsychological features”, “therapeutics”).
2. Selection Criteria: We included peer-reviewed original research articles, review articles, and clinical guidelines that contributed significantly to the understanding of PPA. Although we did not apply strict inclusion and exclusion criteria as in a systematic review, our selection focused on studies offering relevant clinical insights and comprehensive data on the subject.
3. Data Extraction and Synthesis: Relevant information regarding clinical features, diagnostic challenges, and management strategies was extracted from the selected studies. The data were then synthesized qualitatively through an iterative process among the authors to provide a broad, clinically relevant narrative that highlights current concepts, controversies, and future directions in PPA research.

We believe that the added value of this work lies in its comprehensive synthesis of current knowledge—from epidemiological trends to clinical presentations and diagnostic challenges. By integrating findings from a broad range of studies, our review offers clinicians a cohesive and accessible overview of PPA, highlighting key controversies and gaps in the literature. This synthesis not only informs clinical practice but also lays the groundwork for future research, including more focused systematic reviews. As such, we will start with the following brief historical background on the study of PPA.

## 2. Historical Overview

The most significant findings regarding Primary Progressive Aphasias (PPAs) have been documented over time. In 1892, Pick provided the first description of a language disorder of degenerative origin, attributed to frontotemporal atrophy [2]. Building on these observations, Mesulam later characterized a “slowly progressive aphasia without generalized dementia” [3], emphasizing a distinct neurodegenerative pathology: PPA. This condition is typically associated with progressive focal atrophy of the left perisylvian regions of the dominant hemisphere [1].

In 1998, Neary proposed that PPA should be considered secondary to pathologies unrelated to Alzheimer’s disease, integrating it into the spectrum of frontotemporal lobar degeneration (FTLD). Subsequently, two distinct forms of PPA were identified: primary progressive non-fluent aphasia and semantic dementia [4]. These milestones underscore the evolving understanding of PPA as a unique and specific pathology within neurodegenerative disorders.

Mesulam proposed diagnostic criteria for PPA, establishing that this pathology is characterized by a language deficit as the initial symptom (Table 1). Neurodegenerative language disorders were first characterized according to two forms: progressive non-fluent and fluent aphasia [1]. However, Gorno-Tempini et al. [5] described in 2004 a third type of PPA, primary progressive logopenic aphasia, which is most often associated with a focal form of Alzheimer’s disease (AD) [6]. In 2011, the same authors [7] proposed an updated classification of PPA, incorporating three distinct subtypes: non-fluent/agrammatic PPA, semantic PPA (or semantic dementia), and logopenic PPA. This classification was based on the correlation between clinical features, patterns of brain atrophy, and the use of biomarkers to identify underlying pathologies. This new framework has since guided research and diagnosis, providing a clearer understanding of the distinct clinical and pathological features of PPA subtypes.

## 3. Diagnostic Criteria

**Diagnostic Criteria for PPA According to Mesulam [1]**

Gorno-Tempini et al. [7] proposed updated general criteria for the diagnosis of Primary Progressive Aphasia (PPA), building on and expanding Mesulam’s original criteria [1] (see Table 1 and Table 2). These new criteria are less restrictive than those outlined by Mesulam. For instance, Gorno-Tempini introduced the broader concept of a “language complaint” without specifically requiring the detailed language impairments mentioned by Mesulam, such as anomia or spontaneous language comprehension disorders.

Another notable difference is the minimum duration for isolated aphasia: Gorno-Tempini does not stipulate a required duration before the onset of other cognitive disorders, whereas Mesulam mandates that aphasia remains isolated for at least two years. Additionally, while language disorders remain the primary driver of reduced daily functioning, Gorno-Tempini acknowledges that they are not the sole factor affecting activities of daily living. Lastly, previous linguistic ability is no longer considered an exclusion criterion in Gorno-Tempini’s updated framework, making it more inclusive and adaptable for diverse clinical scenarios.

## 4. Epidemiology

PPA cases have been reported globally, with no clear geographic clustering. Studies show that PPA affects diverse populations, although prevalence estimates are similar across regions, which are estimated to range between 3 per 100,000 [8] and 7 per 100,000. In Memory Clinics, 0.5–2.5% of patients diagnosed with a neurodegenerative disease are estimated to have PPA. The overall impact of PPA in the general population is approximately 1 per 100,000 [9]. Variations in prevalence and subtype distribution might reflect differences in the awareness and diagnosis of PPA, especially in resource-limited settings where neuroimaging and specialized cognitive evaluations are less accessible.

PPA typically begins around the age of 60, with an onset range between 45 and 70 years. The disease duration varies widely, from 4 to 14 years, with an average duration of approximately 8 years [10]. Gender prevalence differs depending on the study. Some findings suggest PPA is more frequent in men than in women [1], with a reported distribution of 66% men and 34% women [11]. Another study found a less pronounced male predominance, reporting 45 men and 40 women among 85 patients [12]. Contrarily, Kertesz [13] highlighted a predominance of PPA in women, with 60% of patients being female. While there does not appear to be a definitive sex ratio, some studies suggest subtype-specific differences, with a male predominance in semantic variant PPA and a female predominance in non-fluent/agrammatic variant PPA [12]. Additionally, after the age of 80, there is a notably higher prevalence of PPA in men [8], similar to what is observed in limbic-predominant age-related TDP-43 encephalopathy (LATE).

Certain neurodevelopmental factors may increase the risk of developing PPA [14]. For instance, individuals with language development disorders such as dyslexia are more frequently diagnosed with PPA, particularly the logopenic variant. This may be attributed to shared cognitive (phonological deficits) and anatomical (posterior temporoparietal cortex involvement) substrates between dyslexia and logopenic PPA [15]. PPA may thus reflect the late manifestation of a vulnerability in linguistic networks, which could also explain the male predominance in some PPA populations [8].

A positive family history of neurodegenerative diseases is reported in 20–40% of PPA patients, suggesting a role for genetic factors [9]. To date, however, no significant environmental risk factors have been identified in the literature [16].

The limited representation of non-Caucasian populations in PPA research highlights the need for broader, more inclusive epidemiological studies to clarify whether any demographic or racial disparities exist.

## 5. Genetics of PPA

The genetic background of Primary Progressive Aphasia (PPA) is not entirely clear, but a subset of cases does appear to have a genetic component, especially those associated with frontotemporal lobar degeneration (FTLD). The likelihood of a genetic influence depends on the PPA subtype and underlying pathology.

PPA is a neurodegenerative disease in which at least three main types of underlying conditions are found: FTLD-tau, FTLD-TDP-43, and AD. Author E. Finger [17] estimated that around 40% of cases are associated with a familial inheritance pattern, but only 10% have an autosomal dominant inheritance pattern. Although the vast majority of PPA cases are sporadic, few familial cases of PPA with pathological variants in the genes associated with FTLD have been reported: microtubule-associated protein tau (MAPT), granulin precursor (GRN), and C9orf72, as well as in the presenilin 1 (PSEN1) gene, which causes early onset AD. In the familial cases studied so far, genealogical characteristics consistent with an autosomal dominant inheritance pattern have been found [18].

In a study published by the Mesulam working group [19], where targeted sequencing was performed for the main genes responsible for both AD and frontotemporal dementia (APP, TARDBP, FUS, GRN, MAPT, PSEN1, PSEN2, C9orf72, MAPT A152T, TREM2 R47H, APOE) in 403 cases of APP, they apparently found that 14 (3.5%) cases were carriers of pathological variants: 4 showed expansions of the C9orf72 gene, 9 in the GRN gene, and 1 in the TAR DNA-binding protein (TARDBP) gene. Regarding the risk genes, they identified 114 carriers of at least one ε4 allele, of which 14 were ε4-ε4 (6 e2-ε4 and 94 ε3-ε4). In turn, they found 273 (67.7%) carriers of the H1-H1 haplotype of the 17q.21.31 haplotype, (MAPT/tau haplotype), and 114 (28.3%) and 16 (4%) had an H1-H2 and H2-H2 haplotype, respectively.

In a systematic review published by our group [20], Orozco-Barajas described the neuropsychological changes in carriers of the PSEN1 A431E variant, along with other genetic mutations. Among individuals with mild cognitive impairment carrying both a PSEN1 and APP mutation, lower fluency scores were observed compared to preclinical carriers and controls. Additionally, unpublished data from our group have also shown reduced fluency scores in carriers of the PSEN1 A431E variant.

While a clear genetic background exists for some cases of PPA, particularly those tied to FTLD, the majority are sporadic. Genetic testing is generally recommended in individuals with a strong family history of neurodegenerative diseases or in cases suggestive of familial FTLD. Understanding the genetic basis of PPA can help guide diagnosis, prognostics, and potential future therapies.

## 6. Neuropathology and Discovery of TDP-43

Although clinical and neuropathological diagnoses of Alzheimer’s disease (AD) coincide in approximately 90% of cases at specialized centers, predicting the underlying pathology of Primary Progressive Aphasia (PPA) is more challenging due to its histopathological heterogeneity. In terms of classifying diseases by proteins with conformational alterations, TDP-43 (TAR DNA-binding protein) proteinopathies and tauopathies are the most common. The recent identification of pathological TDP-43 in the largest histopathological group—frontotemporal lobar degeneration with ubiquitin-positive inclusions (FTLD-U)—represents a significant advance in the emerging research on PPA.

TDP-43 is a nuclear protein expressed in multiple tissues, including the heart, lungs, liver, muscles, and brain. In FTLD-U, abnormal TDP-43 inclusions are found in neurons and glial cells of the cerebral cortex, granule cells of the dentate gyrus, the hippocampus, and the anterior horn cells of the spinal cord. These inclusions, which may also occur in the nucleus, are abnormally phosphorylated and ubiquitinated [21]. Ubiquitin is a protein present in all eukaryotic cells that modifies other proteins, including TDP-43, through a process known as ubiquitination—the “tagging” of target proteins for degradation [22].

**Figure 1:**
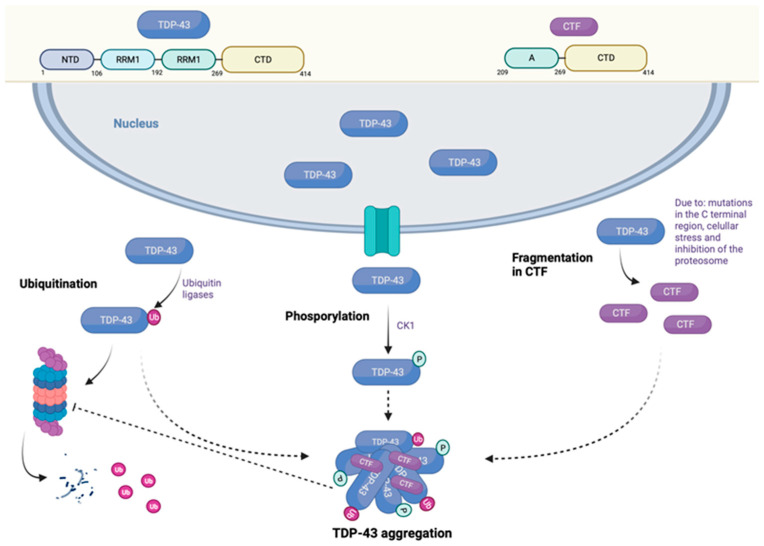
Characteristics of TDP-43, ubiquitination and phosphorylation process and its pathological inclusions. Created in BioRender.

Although the physiological significance of TDP-43 remains unclear, it is known to play a role in transcriptional regulation and exon shunting. While the gene encoding TDP-43 is located on chromosome 1, mutations in several other genes can lead to pathological inclusions of TDP-43 (see Figure 1).

Until recently, it was widely accepted that there was no correlation between certain PPA syndromes and their neuropathological diagnoses. However, recent studies have suggested a different perspective. For instance, while FTLD-U (TDP-43 proteinopathy) is frequently observed in semantic dementia, other tauopathies may be present in mixed and non-fluent forms of PPA. The question of whether logopenic and mixed forms of PPA are primarily variants of Alzheimer’s disease remains controversial. This complexity and the unresolved issues surrounding PPA underscore the critical importance of post-mortem brain examinations in specialized centers.

## 7. Neuroimaging and Neuropathological Features of PPA

**Figure 2:**
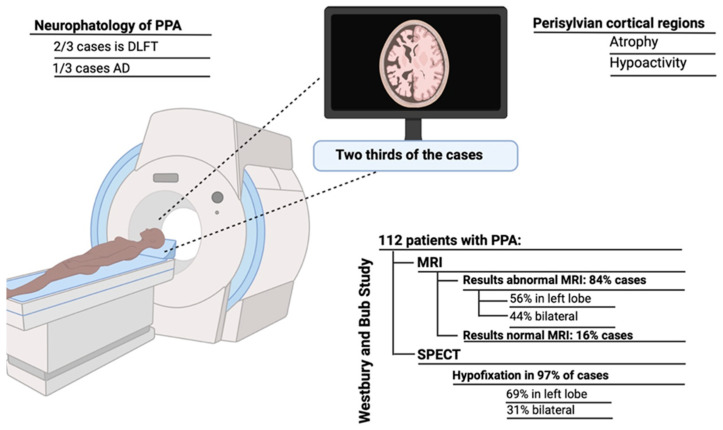
The main neuroimaging studies are MRI and SPECT, with predominant damage in the left lobe and bilaterally. Created in BioRender.

Neuroimaging reveals bilateral atrophy and hypoactivity of the perisylvian cortical regions in approximately two thirds of cases [10]. Westbury and Bub [11] conducted a literature review that included 112 patients with PPA, finding abnormal magnetic resonance images (MRIs) in 84% of cases—56% with abnormalities confined to the left lobe and 44% with bilateral involvement. Additionally, SPECT examinations revealed hypofixation in 97% of cases, with 69% predominantly in the left lobe and 31% bilaterally (Westbury & Bub, 1997 [11]) (see Figure 2). Neuropathologically, PPA is associated with frontotemporal lobar degeneration (FTLD) in approximately two thirds of cases, while Alzheimer’s disease (AD) accounts for one third of cases [10].

The following are the associated brain regions and network implications described for the different types of APP:

**Non-fluent/Agrammatic Variant (nfvPPA)**

Inferior frontal gyrus (Broca’s area): Essential for speech production and grammatical processing.

Supplementary motor area: Plays a critical role in motor planning for speech.

Insular cortex: Contributes to motor control and coordination of speech articulation.

Network Implications:

Disruption of the dorsal language network, which facilitates communication between frontal and posterior regions of the brain.

Key affected pathways include the arcuate fasciculus and superior longitudinal fasciculus, both of which are crucial for syntactic processing and fluent speech production [23].

**Semantic Variant (svPPA)**

Anterior temporal lobe (ATL): Particularly the left ATL, which plays a critical role in semantic processing.

Bilateral ATL involvement: Frequently observed, with atrophy typically more pronounced on the left side.

Network Implications:

Disruption of the ventral language network, which includes the following:

Inferior longitudinal fasciculus: Connects the occipital and temporal lobes, supporting semantic processing.

Uncinate fasciculus: Links the temporal lobe with the frontal regions for semantic integration.

Impairment of networks involved in object recognition and conceptual knowledge, leading to difficulties in understanding the meaning of words, objects, and concepts [24].

**Logopenic Variant (lPPA)**

Posterior superior temporal gyrus—critical for phonological processing.

Inferior parietal lobule, particularly the angular gyrus—involved in language and working memory.

Network Implications:

Disruption of the dorsal language network, particularly connections between temporal and parietal regions via the arcuate fasciculus.

Impaired working memory and phonological loop function, which are crucial for sentence repetition and word retrieval [25].

## 8. Evolution of Disorders and Prognosis

The duration of disease progression varies considerably across studies, ranging between 5 and 10 years. Disruptions in daily life typically occur on average 6 or 7 years after the onset of initial symptoms, with a range of 2 to 12 years [26]. In terms of affected domains, PPA primarily impacts executive function, speech production (often leading to mutism), action planning, and language comprehension, with less pronounced effects on emotional regulation and behavior. Conversely, non-verbal and verbal memory, calculation, and visuospatial representation are generally less affected [27]. At more advanced stages, there is a marked deterioration in language abilities, particularly in production and comprehension [1]. Additionally, global cognitive and behavioral impairments may emerge, leading to significant disruptions in daily living activities and a considerable loss of patient autonomy [28].

Regardless of the initial form of PPA, the progression generally tends toward global aphasia, characterized by mutism and severe comprehension deficits [1]. Although mutism is typically a late-stage feature in dementia syndromes, it may appear early in PPA, even when other cognitive functions remain relatively preserved [14]. In more advanced stages—marked by deficits in additional cognitive domains or the emergence of motor impairments—Mesulam recommends the term PPA+ to indicate that PPA is no longer the sole defining feature of the patient’s phenotype [2].

In non-fluent PPA, the progression often leads to executive deficits, apraxia of speech, and abnormal movements associated with corticobasal syndrome or progressive supranuclear palsy, with atrophy extending to the dorsolateral prefrontal cortex, motor areas, and basal ganglia [2]. In semantic PPA, disease evolution may result in behavioral disorders and associative agnosias when atrophy spreads to the insular and orbitofrontal cortices and the contralateral anterior temporal lobe [2]. The progression of logopenic PPA is the most variable among the PPA subtypes; in some patients, the logopenic pattern may represent a prodromal stage of non-fluent or semantic PPA, while in others, atrophy progressing toward the medial temporal lobe results in episodic memory deficits. This variant of PPA may also be associated with Alzheimer’s disease [2].

**Specificities of the disorders**

The classification of PPA [7] is based on the correlation between clinical criteria and additional examinations (imaging and biomarkers). Three subtypes of PPA are described (Table 3):

- Non-fluent PPA;
- Semantic PPA;
- Logopenic PPA.

**Figure 3:**
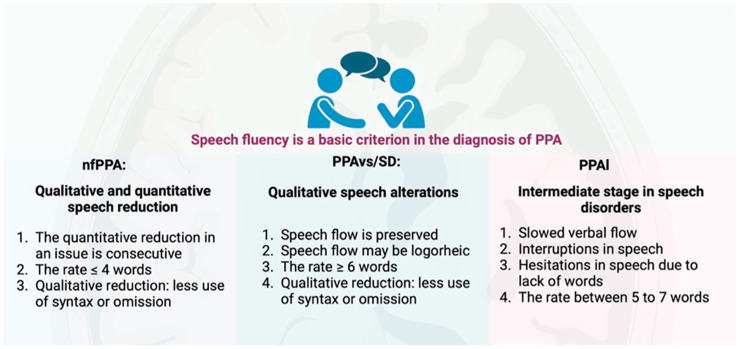
Speech fluency criteria for the diagnosis of PPA. Created in BioRender.

Part of the diagnosis of PPA relies on the fluency criterion. Non-fluent aphasias are characterized by both quantitative and qualitative reductions in speech. Quantitatively, this reduction is reflected in a lower average number of consecutive words produced in a single utterance (typically ≤4 words). Qualitatively, it involves a reduction in or even omission of syntactic structures. In contrast, semantic aphasia is marked by impaired language quality while maintaining a preserved flow, which can sometimes be logorrheic, with a fluency of ≥6 words per utterance. Logopenic aphasias represent an intermediate stage, featuring a slowed verbal flow due to frequent interruptions and hesitations—primarily resulting from word retrieval difficulties—with fluency typically ranging between five and seven words per utterance [29] (Figure 3).

**Non-fluent Primary Progressive Aphasia (nfPPA)**

In various studies, PPA is diagnosed in approximately 45% of FTLD cases, with non-fluent PPA (nfPPA) accounting for 50% of these. The prevalence of nfPPA due to FTLD ranges from 0.5 to 3 per 100,000, with an incidence of 0.4, to 0.7 per 100,000 per year; in contrast, PPA attributed to AD has a prevalence of 0.7 to 3.9 per 100,000 and an incidence of 0.5 to 0.9 per 100,000 per year [30] (see Table 4). Moreover, an imaging-supported diagnosis should fulfill both criteria outlined in Table 4.

**Factors related to reduced fluency**

Agrammatism is characterized by the production of syntactically simplified sentences. Patients tend to produce short utterances that omit grammatical morphemes—such as pronouns, prepositions, and markers of gender and number—and make frequent grammatical errors. This speech pattern is often described as “telegraphic” due to its abbreviated nature, though it generally remains informative [31].

**Figure 4:**
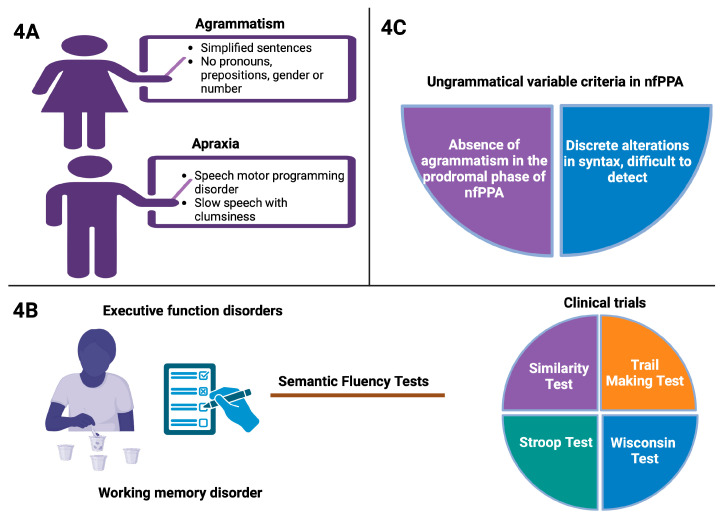
(**A**) Differences between agrammatism and apraxia. (**B**) Main clinical evaluations to analyze alterations in executive functions and working memory. (**C**) Differences in the evaluation of agrammatic criteria in nfPPA. Created in BioRender.

Apraxia of speech, in contrast, is a disorder of speech motor programming [10]. Affected individuals exhibit labored, strained speech accompanied by phonetic distortions and phonemic errors. Their speech flow is slowed, with observable articulatory clumsiness, false starts, frequent restarts, self-corrections, and silent pauses. Additionally, there is considerable variability and inconsistency in these deficits over time, reflecting an automatic voluntary dissociation typical of apraxia of speech [32] (see Figure 4A).

In executive function and working memory disorders, clinical pathological data are obtained from various tests, including semantic and letter fluency, the similarity test, the Trail Making Test, Stroop, and the Wisconsin Card Sorting Test [33] (see Figure 4B). Some authors attribute difficulties in understanding complex sentences to a deficit in working memory [34]. The prevalence of the agrammatic criterion in nfPPA varies considerably across studies [34]. One hypothesis suggests that frank agrammatism may be absent in the prodromal phase and develop later in the disease course [35]. Syntax alterations—such as syntactic simplification and reduced utterance length—are typically subtle [36] and challenging to detect using both qualitative (spontaneous language analysis) and quantitative assessments (see Figure 4C). This subtlety could explain the lack of significant differences in outcomes between nfPPA patients and control populations [35].

Neuropsychiatric disorders are uncommon at the onset of non-fluent primary progressive aphasia (nfPPA) (Macoir et al., 2017 [33]). However, several authors have noted the presence of behavioral disturbances, including apathy in 42% of nfPPA cases and loss of empathy in 40% (Van Langenhove, Leyton, Piguet, & Hodges, 2016 [37]). This loss of empathy, which impairs the recognition of feelings and emotions in others, may serve as a useful differential diagnostic marker between nfPPA and logopenic PPA, where it is observed in only 13% of cases. Additionally, agitation and depression have been reported in subjects with nfPPA [33].

**Primary progressive fluent/semantic variant aphasia (svPPA)**

Approximately 25% of FTLD cases meet the criteria for primary progressive fluent aphasia [38], with the prevalence of this pathology estimated at 0.8 per 100,000 (Eloi Magnin et al., 2016 [8]). Fluent PPA is the variant that has achieved the greatest consensus in its description [39]. Originally, the term “semantic dementia” (SD) was used to describe the loss of knowledge related to objects, places, people, and concepts [38]. In practice, however, it has been applied as part of semantic PPA to denote the loss of word meaning. Recent classifications propose grouping pathologies associated with semantic memory disorders into two categories—those with multimodal impairment (semantic dementia) and those with an isolated verbal deficit (unimodal impairment, often referred to as APP)—under the common term “semantic PPA”. This grouping is justified by factors such as the involvement of similar brain structures and the presence of common histopathological lesions (see Table 5). Single-word comprehension deficits, particularly for unfamiliar and low-frequency words, are a hallmark of this variant [10]. Moreover, neuropsychiatric disorders tend to be more prevalent and appear earlier in semantic PPA, with behavioral manifestations like those seen in the behavioral variant of frontotemporal dementia, including stereotyped behavior (50%), dietary changes (22%), disinhibition (17%), apathy (11%), and loss of empathy (9%) [37].

In recent years, an asymmetric temporal lobe presentation has been described in cases of frontotemporal lobar degeneration (FTLD), with the involvement of the left temporal lobe being up to three times more common than the right [40]. A distinct right temporal variant of frontotemporal dementia (rtvFTD) has been identified and is considered a type of svPPA. At onset, semantic loss is more frequently observed in cases involving left temporal lobe FTLD, whereas behavioral changes are more commonly associated with rtvFTD. In a recent systematic review performed by Ulugut, H. et al. (2021) [41], FTLD-TDP type C was the most common underlying pathology in rtvFTD. In up to 64% of rtvFTD, underlying pathologies other than FTLD-TDP type C were present, such as Tau-MAPT and FTLD-TDP type A and B [41].

**Primary progressive logopenic aphasia (lPPA)**

The proportion of patients with lPPA varies between 32% and 52%, depending on the study [8]. Several elements allow us to differentiate logopenic PPA from the other two variants [7]:

The relative preservation of grammatical skills, the absence of speech disorders, motor programming, prosody.

The preservation of semantic memory. The observed deficits can be attributed to impairments in short-term memory and auditory–verbal working memory. Specifically, dysfunction of the phonological loop—which encompasses both the phonological store and the subvocal rehearsal system—appears to underlie many of these impairments [42]. This dysfunction explains the difficulties in comprehending and repeating sentences, particularly longer ones (a length-dependent deficit; see Table 6). Notably, this impairment is more severe in logopenic PPA than in other PPA variants [8]. Additionally, patients with lPPA exhibit poor performance in tests of episodic memory, calculation, and executive function [8]. Anxiety and agitation are frequently observed, and neuropsychiatric symptoms such as stereotyped behavior (14%) and loss of empathy (13%) have also been reported [37].

## 9. Assessment of Primary Progressive Aphasias

**Sociodemographic characteristics of patients with PPA**

The sociodemographic characteristics of patients diagnosed with PPA relate to the age of onset of the disorders, the age of diagnosis, the level of education, and the subjects’ lifestyle. In a retrospective study of 112 cases, the mean age of onset of the disorders was 59 years. The average age of diagnosis was 63.4 years [11]. A second retrospective study carried out between 1992 and 2001, which included 49 cases, proposed an age of diagnosis of around 66 years (52–80) [26]. The time between the onset of the disorders and the announcement of the diagnosis is 4 years for both studies. According to some studies, the diagnoses of nfPPA and lPPA are the most difficult to make, particularly at the beginning of the disease [6,7]. In this study, the level of confidence of professionals when making the diagnosis is measured using a scale from 0 to 10. These are nfPPA and lPPA [8]. This could explain the results found in the study by Magnin et al. [8], where patients with nfPPA and lPPA are relatively older than patients with semantic PPA.

**Figure 5:**
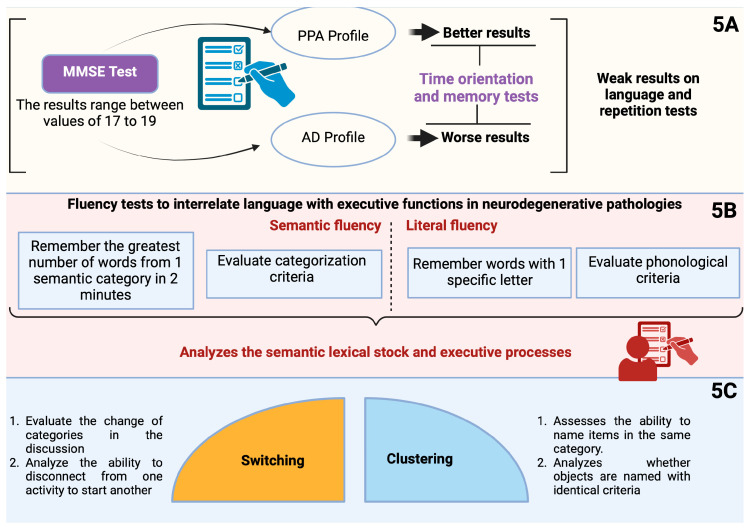
(**A**) MMSE testing in patients with PPA and AD. (**B**) Differences in fluency tests. (**C**) Characteristics of switching and clustering. Created in BioRender.

The mean Mini Mental State Examination (MMSE) scores reported in various studies have been 17 [26], 18 [43], and 19 [44]. However, the utility of the MMSE in this population may be limited, as language disorders—whether in production or comprehension—can influence performance on neuropsychological assessments [14]. Specific tests have proven valuable for the early identification of disorders and in facilitating differential diagnosis. Notably, the MMSE reveals distinct profiles when comparing PPA to Alzheimer’s disease (AD): patients with PPA generally perform better on temporal orientation and memory tasks (such as the three-word recall) than those with AD, yet they show weaker performance on language and repetition tests [14] (see Figure 5A).

Results obtained from the patient’s first consultation are a good predictor of the preservation of autonomy during the disease [26]. Fluency tests are particularly sensitive to the interrelation between language and executive functions in neurodegenerative pathologies [45].

They are of two types:

- Semantic or Categorical Fluency Tasks:

Patient is asked to generate as many words as possible that belong to a specific semantic category (e.g., animals, fruits) within a limited time frame (typically one to two minutes).

- Letter Fluency Tasks:

Subject is asked to recall as many words as possible that begin with a given letter (see Figure 5B).

These two tests allow for the evaluation of the stock, access to the lexical-semantic stock, and executive processes involved in the initiation, the organization of the search, and the verbal production or even the inhibition of already given or irrelevant answers [45]. In the semantic fluency task, the search is carried out using categorization criteria, while in letter fluency, the access is carried out using phonological criteria [46].

The study by Raoux et al. [46] carried out a qualitative analysis of the results focusing on the strategies used by the patients during this test. It highlights two processes:

- Switching (changing categories during the discussion, ability to disconnect from one activity to invest in another). This is the case, for example, when the patient switches from words beginning with PA to words beginning with PO.
- Clustering (the production of articles that can be grouped under identical criteria). For example, the patient puts together all the names of pets to think about (Figure 5C).

The authors demonstrated that, for subjects with dementia, the number of switches decreases beginning in the prodromal phase (approximately five years before diagnosis), even while overall quantitative performance remains relatively intact [46]. A subsequent study indicated that both verbal and non-verbal graphic fluency tests—such as the Ruff Figural Fluency Test—could serve as specific indicators for PPA [47,48]. Specifically, patients with semantic variant PPA (sPPA) exhibit a significant deficit in categorical fluency tasks, whereas those with non-fluent variant PPA (nfPPA) show greater impairment in letter fluency than in categorical fluency. In contrast, patients with logopenic PPA (lPPA) present a relatively uniform deficit across both tests. Additionally, non-verbal fluency is particularly compromised in nfPPA patients, who display a marked initiation deficit [47,48].

## 10. Diagnosis and Treatments

Characterizing the PPA syndrome is challenging due to its semiological heterogeneity and the progressive nature of the deficits [28]. Moreover, some authors note that cases with an isolated language disorder persisting over a long period are rare [26]. Confirming the diagnosis requires evaluation at specialized memory centers, where additional examinations—such as biomarker analysis, detailed speech therapy assessments, and comprehensive neuropsychological tests—can be performed [8]. FTLD pathology underlines all subjects with nfPPA [49], other progressive profiles, including Alzheimer’s disease, diffuse Lewy body disease, and corticobasal degeneration, have also been described [14].

Currently, there is no validated pharmacological treatment for PPA. Some antidementia drugs—such as bromocriptine, galantamine, and donepezil—have been tested in small cohorts, and while some positive effects have been suggested [14,28], their efficacy remains uncertain. Additionally, patients’ relative awareness of their deficits can lead to depressive symptoms, warranting the prescription of antidepressants [28]. The application of transcranial magnetic stimulation (TMS) also requires further validation, as existing studies have involved an insufficient number of cases [50]. To address this limitation, a recent meta-analysis involving 513 AD patients treated with repetitive TMS demonstrated significant improvements in daily living activities, although no notable benefits were observed in cognitive domains such as memory, language, or executive function [51]. Moreover, it is important to note that acetylcholinesterase inhibitors may exacerbate some behavioral symptoms [14].

**Figure 6:**
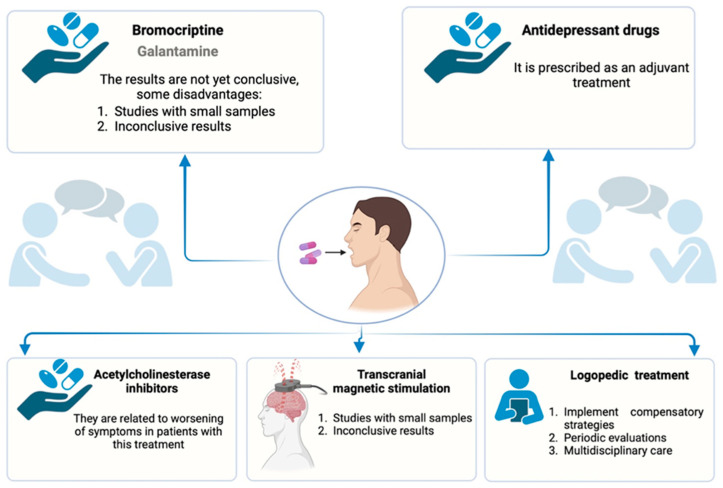
Different treatments in patients with PPA. Created in BioRender.

In the absence of a specific pharmacological treatment, speech therapy can be offered to optimize the patient’s residual capacities and implement compensatory strategies [14]. Although there is no consensus on the optimal approach to speech therapy, the pace and frequency of sessions should be tailored to the individual patient and the progression of the disease [52]. Regular, periodic assessments are essential for monitoring disease progression and ensuring that rehabilitation remains appropriate [31]. Importantly, speech therapy should be integrated into a multidisciplinary care approach, with close collaboration from the patient’s support network [44] (see Figure 6).

In a recent paper by Antonioni et al. [53], they demonstrated that blood-based pTau assays can accurately reflect AD-related pathological changes. This is especially relevant for the logopenic variant of Primary Progressive Aphasia (PPA), which is frequently associated with AD pathology. The integration of blood-based pTau into clinical practice offers several important implications.

Enhanced Diagnostic Precision:

Blood-based pTau provides an objective, minimally invasive biomarker that can complement neuropsychological evaluations and imaging studies. Studies such as those by Karikari et al. [54] and Janelidze et al. [55] have shown that plasma pTau181 reliably distinguishes AD pathology. This is particularly crucial for differentiating AD-related PPA from other neurodegenerative conditions, where clinical presentations may overlap.

Early Identification and Treatment Stratification:

The early detection of AD pathology via blood-based pTau assays facilitates timely therapeutic interventions. Research by Palmqvist et al. [56] indicated that early biomarker identification can be instrumental in guiding treatment strategies. By identifying patients with logopenic PPA who exhibit AD biomarkers, clinicians can implement targeted, potentially disease-modifying treatments at an earlier stage.

Clinical and Research Implications:

Highlighting the reliability of blood-based pTau not only reinforces its current role in clinical diagnostics but also encourages further exploration of its applications. As the field moves toward personalized medicine, such biomarkers could underpin more nuanced therapeutic approaches tailored to individual pathological profiles. This aligns with our goal of providing a comprehensive overview of PPA and its relationship with neurodegenerative diseases.

Recent advancements in Alzheimer’s disease (AD) therapeutics have led to the development of several novel agents that hold promises for refining both the differential diagnosis and treatment of Primary Progressive Aphasia (PPA), particularly in cases where AD pathology underlies clinical presentation. We list these agents as follows:

Aducanumab: Aducanumab, an amyloid-beta monoclonal antibody, was initially developed and even approved in certain regions for AD treatment [57,58]. However, due to controversies regarding its clinical efficacy and safety, its use has been discontinued in some markets. Despite this, the development of aducanumab has significantly contributed to our understanding of anti-amyloid strategies and has paved the way for the exploration of alternative therapies for AD-associated conditions, which could be the case for the logopenic variant of PPA.

Lecanemab: A newer anti-amyloid agent, Lecanemab targets soluble amyloid aggregates. Early clinical trial data indicate that it can reduce amyloid levels and may offer therapeutic benefits for AD-related PPA [59].

Donanemab: Donanemab is another monoclonal antibody targeting amyloid plaques, and is currently under investigation. Its potential to modify disease progression could help in identifying patients with an AD profile among those with PPA [60].

Tau-Targeting Therapies: Several investigational drugs focus on reducing tau pathology—a hallmark of AD. Anti-tau therapies, including monoclonal antibodies against tau, are in various clinical development stages and may be beneficial for PPA variants associated with tau abnormalities [61,62].

Other Investigational Agents: Ongoing research is also targeting neuroinflammation, synaptic dysfunction, and oxidative stress, all of which are implicated in AD pathology. These emerging therapies may broaden the treatment options for patients with AD-associated PPA [58].

Implications for Differential Diagnosis:

Biomarker-Driven Differentiation:

The availability of these novel AD drugs reinforces the utility of biomarkers—such as blood-based pTau, CSF amyloid-beta, and tau imaging—in differentiating AD-related PPA from other PPA subtypes.

Therapeutic Trials as Diagnostic Tools:

Observing a patient’s response to these novel therapeutics may provide indirect evidence of underlying AD pathology. A favorable response to anti-amyloid or anti-tau treatments could reinforce the diagnosis of logopenic PPA as being driven by AD pathology.

Implications for PPA Therapeutics:

Tailored Treatment Approaches:

Incorporating novel AD drugs into the management of PPA could lead to personalized treatment protocols. Patients with AD biomarkers presenting with PPA might benefit from these targeted agents, potentially slowing disease progression and improving functional outcomes.

Future Research Directions:

Further studies and clinical trials focusing on PPA populations are needed to evaluate the efficacy and safety of these novel agents in this specific group. This research will be crucial in refining both differential diagnosis and therapeutic strategies for PPA.

**Diagnostic classification of PPA**

**Decision tree in PPA (Leyton, 2011)** [63]

There is a decision-making algorithm developed from the Progressive Aphasia Language Scale (PALS). This scale allows for the classification of 45 of the 47 PPAs studied, or 96% of them [63]. The authors show that four items are discriminatory for the diagnosis of PPA subtypes (disorders of motor programming of speech, agrammatism, comprehension of isolated words, repetition of sentences). This decision tree is compatible with the classification proposed by Gorno-Tempini and allows the clinician a quick and clear view of the different clinical profiles.

**Criteria (Vandenberghe, 2016)** [64]

**Figure 7:**
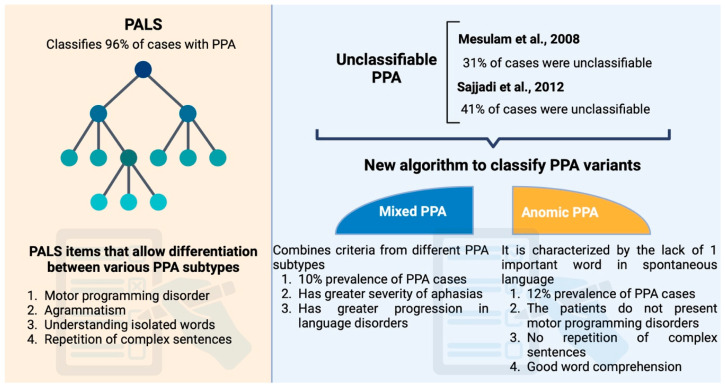
Factors to classify PPA cases using different criteria [49,65]. Created in BioRender.

Several published studies indicate that a subset of PPAs do not fit into any of the currently described subtypes. These cases are termed “unclassifiable” because they fail to meet all the necessary criteria for the three subtypes proposed by Gorno-Tempini [7]. For instance, one study of 84 PPA patients found that 31% were unclassifiable [49], while another study of 46 patients reported that 41% did not satisfy the criteria for any of the three subtypes (see Figure 7) [65]. To reduce the number of unclassifiable cases, Vandenberghe [64] proposed a new decision-making algorithm that incorporates two additional variants of PPA:

- Mixed PPA;
- Anomic PPA.

The combination of criteria from different PPA subtypes constitutes a mixed variant of PPA. The mixed PPA phenotype was introduced in 2014 to describe cases in which patients present comprehension deficits along with agrammatism and/or apraxia of speech [2]. This variant accounts for approximately 10% of PPA cases (Vandenberghe, 2016 [64]) and appears to be of greater concern in patients evaluated at more advanced stages of the disease due to the severity of aphasia and progressive language deterioration [2]. Biological examinations typically reveal atrophy of the inferior frontal cortex as well as the anterior and superior temporal cortices [2]. In cases where patients do not exhibit the distinctive features of the three PPA subtypes—such as motor programming disorders, the impaired repetition of complex sentences, or deficits in word comprehension—the PPA may be described as anomic. This subtype is characterized by the omission of particularly important words in spontaneous language [64], and its prevalence is reported to be approximately 12% (3 in 25 cases) [66].

**Limitations of the classification of PPA**

Certain complex and unclassifiable cases can be grouped under common criteria, forming an additional, clinically relevant PPA subtype. Thus, it is essential for both research and clinical practice to recognize that the three subtypes proposed by Gorno-Tempini do not fully encompass the entire spectrum of the pathology. For instance, this classification largely overlooks communication and written language disorders—except in the case of semantic variant PPA (sPPA). Other variants could be further characterized by evaluating performance on written language tests [67,68]. For example, the phonological deficits present in logopenic PPA (lPPA) often lead to abnormal performance when reading pseudowords [39]. Numerous studies emphasize the need to standardize language assessments for PPAs and to utilize relevant tests that yield comparable and reproducible results, thereby overcoming the limitations of current consensus recommendations [39].

Recent studies have demonstrated the potential of automatic speech analysis algorithms in the identification and classification of Primary Progressive Aphasia (PPA) variants:

- Automated Speech Biomarkers:

Nevler et al. (2018) examined digitized speech samples from native English speakers who met published clinical consensus criteria for a specific PPA syndrome and showed that analyzing acoustic properties of speech, such as prosody, can help distinguish between PPA variants [69].

- Mobile and Wearable Speech Monitoring Apps:

While specific studies on mobile applications for PPA are limited, the advancement in automatic speech recognition and analysis suggests potential for developing such tools for continuous monitoring [70].

- Multimodal AI Systems:

Combining speech analysis with other modalities, such as neuroimaging, has been proposed to enhance the classification of PPA subtypes [71].

These studies highlight the promise of integrating automatic speech analysis algorithms into clinical practice for earlier and more accurate diagnosis of PPA.

**Differential Diagnosis**

The most common differential diagnosis for PPA is Alzheimer’s disease (AD). In progressive fluent aphasia, decreased verbal comprehension can mimic memory impairment on verbal memory tests. Conversely, a disorder of the semantic-lexical system is often present in AD. However, the marked semantic impairment in PPA, combined with the absence of true amnesia and the presence of functional and morphological parietal brain alterations, typically allows for differentiation from AD. Simple screening tests such as the Mini Mental State Examination (MMSE) or the Montreal Cognitive Assessment (MoCA) can aid in this distinction. For instance, patients with PPA tend to have greater difficulty encoding three nouns, naming objects, following a three-step command, and repeating function words, yet they show relatively fewer problems remembering a list of three words or copying two pentagons—although these differences are not statistically significant when compared to AD patients [72]. Moreover, a study by Wood from the Mesulam group found that, when using the MoCA, patients with PPA demonstrated poorer performance in language and attention, whereas deficits in memory and orientation were more pronounced in individuals with Alzheimer-type dementia [73].

Non-fluent progressive aphasia can occasionally serve as the initial manifestation of corticobasal degeneration. In this neurodegenerative condition, unilateral or asymmetric hypokinetic-rigid symptoms are typically associated with cortical signs on the side of the most affected hemisphere. Similarly, progressive supranuclear palsy (PSP) may be accompanied by progressive non-fluent aphasia. The classic features of PSP include predominantly axial parkinsonism with recurrent falls, vertical ocular motility disturbances, and fronto-subcortical impairments—such as psychomotor slowing, apathy, and difficulties with planning, comprehension, and transposition [74].

**Figure 8:**
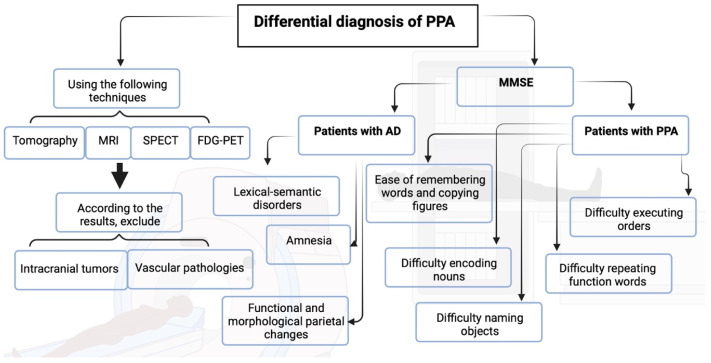
Characteristics of the differential diagnosis of patients in AD and PPA. Created in BioRender. Torres, D. (2024).

To make the diagnosis of PPA, it is essential to exclude other slowly progressive etiologies, especially intracranial tumors and vascular pathologies, either by computed tomography (CT) or MRI. In particular, MRI can provide positive clues about this disease: in progressive non-fluent aphasia, an atrophic enlargement of the left Sylvian fissure is typical, whereas for semantic dementia, atrophy of the left temporal lobe is especially typical. In the absence of atrophy, or if the differential diagnosis presents difficulties, the demonstration by nuclear medicine (CBF-SPECT or FDG-PET) of a typical localized and circumscribed decrease in activity can be useful to support the diagnosis of PPA (Figure 8).

**Prodromal Symptoms of PPA**

In view of the data, we consider that prodromal symptoms of Primary Progressive Aphasia (PPA) are subtle and often overlooked, but emerging research suggests that certain early signs can indicate a risk for developing the disorder. These may include mild language difficulties, such as word-finding problems, reduced fluency, or subtle comprehension deficits, which may progress to full-blown PPA depending on the subtype. Phonological deficits, such as difficulty repeating long or complex words, can also be early indicators, particularly for the logopenic variant (lPPA). Behavioral changes, such as altered social behavior or reduced empathy, may precede the semantic variant (sPPA). Additionally, individuals with a history of developmental language disorders, like dyslexia, may be predisposed to PPA, especially the lPPA form.

Advances in neuroimaging and biomarkers offer promising avenues for early detection. Subclinical atrophy or hypometabolism in regions like the left anterior temporal lobe, posterior temporal–parietal regions, or inferior frontal gyrus may indicate early vulnerability. Biomarkers such as elevated tau, TDP-43, or amyloid-beta in cerebrospinal fluid, along with genetic screening for mutations in GRN, MAPT, or C9orf72, can help identify at-risk individuals. While these early signs and tools are not yet definitive for diagnosis, they may enable earlier interventions in individuals who show prodromal symptoms of PPA.

## 11. Conclusions

PPA is a challenge for diagnosis and treatment; therefore, the characterization and adequate identification of the symptomatology are priorities. Early diagnosis would enable the implementation of therapeutic strategies aimed at slowing disease progression and reducing the severity of symptoms.

Differential diagnosis is essential due to the heterogeneity in both presentation and subtypes of PPA. It is crucial for clinicians to recognize the various linguistic elements that require evaluation to avoid confusing them with memory deficits, as well as with executive or behavioral symptoms. A clear understanding of the variability in clinical presentation allows for greater precision in decision making. Additionally, neuroimaging and genetic studies should be considered as diagnostic aids, helping to identify any underlying pathologies contributing to the clinical presentation.

Transdisciplinary care for PPA can bring greater sensitivity to current classifications, enabling earlier and more accurate diagnoses while improving the quality of life for individuals with this condition.

## Figures and Tables

**Table 1:** PPA diagnostic criteria by Mesulam [1].

PPA Diagnostic Criteria by Mesulam
1 Insidious onset and progressive worsening of loss of or difficulty with understanding through spontaneous speech or formal language tests.
2 Limitation in activities of daily living, different from that generated by language disorders, for at least the first two years following the appearance of the disorders.
3 Normality of premorbid language functions (dyslexia sometimes occurs).
4 Presence during the first two years of apathy, disinhibition, forgetfulness of recent events, visuospatial disorders, visual recognition deficit, and sensorimotor disorders.
5 During the first two years, acalculia or ideomotor apraxia may occur. Discrete constructional apraxias may be observed that do not disrupt daily activities.
6 After the first two years of development, other domains of cognition may be affected. However, language remains the most impaired function, and its impairment profile is faster than in other domains.
7 Exclusion criteria are made through imaging studies of a specific cause such as stroke or tumor.

**Table 2:** PPA inclusion and exclusion criteria ^1^.

PPA Inclusion and Exclusion Criteria
**Inclusion criteria** (Criteria 1–3 must be met)
1 The main characteristic is difficulty with language.
2 Language deficit is the main source of impairment in daily living activities.
3 Aphasia must be the primary deficit at the onset of symptoms and in the initial stages of the disease.
**Exclusion criteria** (Criteria 1–4 must be absent)
1 The pattern of language deficit may be explained by a non-degenerative disease of the nervous system or a medical problem.
2 Cognitive disorders are explained by a psychiatric illness.
3 Initial presence in the foreground of episodic memory disorders, visual memory disorders, and visuoperceptual disorders.
4 Initial foreground presence of behavioral disorders.

^1^ Gorno-Tempini et al., 2011 [7].

**Table 3:** Classification of PPA with clinical criteria, neuroimaging, and biomarkers ^1^.

Classification of PPA
**Non-fluid PPA (nfPPA)**
1 Ungrammatical PPA (G-PPA). PPA non-fluid/ungrammatical
2 Predominant atrophy at the posterior front insular level
3 MRI: Hypoperfusion or hypometabolism predominantly at the left posterior front insular level
**Semantic variant of PPA (svPPA) semantic dementia (SD)**
1 Fluid application
2 Predominant atrophy at the anterior temporal level on MRI
3 And/or hypoperfusion or hypometabolism predominant at the anterior temporal level
**Logopenic PPA (lPPA)**
1 Atrophy predominantly at the perisylvian or parietal level
2 Left posterior damage on MRI
3 And/or predominant hypoperfusion at the level of the left posterior or parietal perisylvium
4 Neuroimaging of the main PPA variants

^1^ Gorno-Tempini et al., 2011 [7].

**Table 4:** Diagnosis of nfPPA ^1^.

Diagnosis of nfPPA
**Clinical diagnosis of nfPPA positive**
At least one criterion must be met
1 Agrammatism
2 Hesitant, labored speech with phonemic errors and distortions (apraxia of speech)
Associated with at least two of the following three signs
1 Problems understanding syntactically complex sentences
2 Preservation of understanding of isolated words
3 Preservation of knowledge about objects
**Neuroimaging studies**
Associated with at least one of the following signs
1 MRI: mainly left posterior frontoinsular atrophy
2 SPECT: hypoperfusion or hypometabolism, mainly left frontoinsular
**Definitive diagnosis of nfPPA**
Definitive clinical diagnosis: associated with at least one of the following signs
1 Neuropathological evidence of a neurodegenerative disease (such as FTLD-tau, FTLD-TDP, AD, others)
2 Presence of a known pathogenic mutation

^1^ Gorno-Tempini et al., 2011 [7].

**Table 5:** Diagnosis of sPPA/SD ^1^.

Diagnosis of svPPA/SD
**Clinical diagnosis of svPPA**
At least two criteria must be met
1 Lack of the word in denomination
2 Problems understanding isolated words
Associated with at least three of the following four signs
1 Loss of knowledge about objects, particularly low-frequency or unknown items
2 Dyslexia or superficial dysgraphia
3 Replay preservation
4 Preservation of the syntax and motor aspects of the language
**Neuroimaging studies**
Associated with at least two of the following signs
1 Clinical diagnosis of svPPA positive
2 MRI: mainly anterior temporal atrophy
3 SPECT: anterior temporal hypoperfusion or hypometabolism
**Positive clinical diagnosis of PPASD**
Definitive clinical diagnosis: associated with one of the following two criteria
1 Neuropathological evidence of a neurodegenerative disease (such as FTLD-tau, FTLD-TDP, AD, others)
2 Presence of a known pathogenic mutation

^1^ Gorno-Tempini et al., 2011 [7].

**Table 6:** Diagnosis of lPPA ^1^.

Diagnosis of lPPA
**Clinical diagnosis of lPPA**
At least two criteria must be met
1 Lack of the word in spontaneous speech and when naming
2 Sentence repetition disorder
Associated with at least three of the following four signs
1 Phonemic paraphasia in spontaneous speech and naming
2 Preservation of the understanding of isolated words and knowledge of objects
3 Preservation of the motor aspects of language
4 Absence of frank agrammatism
**Neuroimaging studies**
Associated with at least two of the following signs
1 Clinical diagnosis of lPPA positive
2 MRI: posterior parietal or perisylvian atrophy with left predominance
3 SPECT: hypoperfusion or posterior parietal or perisylvian hypometabolism, predominantly left
**Positive clinical diagnosis of lPPA**
Definitive clinical diagnosis: associated with one of the following two criteria
1 Neuropathological evidence of a neurodegenerative disease (such as FTLD-tau, FTLD-TDP, AD, others)
2 Presence of a known pathogenic mutation

^1^ Gorno-Tempini et al., 2011 [7].

## Abbreviations

The following abbreviations are used in this manuscript:

PPA	Primary Progressive Aphasia;
FTLD	Frontotemporal Lobar Degeneration;
AD	Alzheimer Disease;
nfPPA	Non-fluent Primary Progressive Aphasia;
PFA	Progressive Fluid Aphasia;
SD	Semantic Dementia;
PMA	Progressive Mixed Aphasia;
MAPT	Microtubule-Associated Protein Tau;
GRN	Granulin Precursor;
PSEN1	Presenilin 1;
APP	Amyloid Precursor Protein;
APOE	Apolipoprotein E;
TARDBP	TAR DNA-Binding Protein;
FUS	FUS RNA-Binding Protein;
PSEN2	Presenilin 2;
C9orf72	Chromosome 9 Open Reading Frame 72;
TREM2	Triggering Receptor Expressed on Myeloid Cells 2;
TDP-43	TAR DNA-binding protein;
FTLD-U	Frontotemporal Lobar Degeneration with Ubiquitin-Positive Inclusions;
MRI	Magnetic Resonance Imaging;
svPPA	Semantic Variant of PPA;
lPPA	Logopenic PPA;
MMSE	Mini Mental Score Examination;
TMS	Transcranial Magnetic Stimulation;
MoCA	Montreal Cognitive Assessment;
CB-SPECT	Cone-Beam Single-Photon Emission Computed Tomography;
FDG-PET	Fludeoxyglucose-18 Positron Emission Tomography;
rtvFTD	right temporal variant of frontotemporal dementia ;
LATE	Limbic-predominant Age-related TDP-43 Encephalopathy.
